# *Haemophilus* is overrepresented in the nasopharynx of infants hospitalized with RSV infection and associated with increased viral load and enhanced mucosal CXCL8 responses

**DOI:** 10.1186/s40168-017-0395-y

**Published:** 2018-01-11

**Authors:** Thomas H. A. Ederveen, Gerben Ferwerda, Inge M. Ahout, Marloes Vissers, Ronald de Groot, Jos Boekhorst, Harro M. Timmerman, Martijn A. Huynen, Sacha A. F. T. van Hijum, Marien I. de Jonge

**Affiliations:** 1 Center for Molecular and Biomolecular Informatics, Radboud Institute for Molecular Life Sciences, Radboud University Medical Center, Nijmegen, The Netherlands; 2 Laboratory of Pediatric Infectious Diseases, Radboud Center for Infectious Diseases, Radboud University Medical Center, Geert Grooteplein-Zuid 10 (Route 412), 6525 GA Nijmegen, The Netherlands; 3 Centre for Infectious Disease Control, National Institute for Public Health and the Environment, Bilthoven, the Netherlands; 4 NIZO, Ede, The Netherlands

**Keywords:** Chemokine, Microbiome, Mucosal inflammation, RSV, Viral load

## Abstract

**Background:**

While almost all infants are infected with respiratory syncytial virus (RSV) before the age of 2 years, only a small percentage develops severe disease. Previous studies suggest that the nasopharyngeal microbiome affects disease development. We therefore studied the effect of the nasopharyngeal microbiome on viral load and mucosal cytokine responses, two important factors influencing the pathophysiology of RSV disease. To determine the relation between (i) the microbiome of the upper respiratory tract, (ii) viral load, and (iii) host mucosal inflammation during an RSV infection, nasopharyngeal microbiota profiles of RSV infected infants (< 6 months) with different levels of disease severity and age-matched healthy controls were determined by 16S rRNA marker gene sequencing. The viral load was measured using qPCR. Nasopharyngeal CCL5, CXCL10, MMP9, IL6, and CXCL8 levels were determined with ELISA.

**Results:**

Viral load in nasopharyngeal aspirates of patients associates significantly to total nasopharyngeal microbiota composition. Healthy infants (*n* = 21) and RSV patients (*n* = 54) display very distinct microbial patterns, primarily characterized by a loss in commensals like *Veillonella* and overrepresentation of opportunistic organisms like *Haemophilus* and *Achromobacter* in RSV-infected individuals. Furthermore, nasopharyngeal microbiota profiles are significantly different based on CXCL8 levels. CXCL8 is a chemokine that was previously found to be indicative for disease severity and for which we find *Haemophilus* abundance as the strongest predictor for CXCL8 levels.

**Conclusions:**

The nasopharyngeal microbiota in young infants with RSV infection is marked by an overrepresentation of the genus *Haemophilus*. We present that this bacterium is associated with viral load and mucosal CXCL8 responses, both which are involved in RSV disease pathogenesis.

**Electronic supplementary material:**

The online version of this article (10.1186/s40168-017-0395-y) contains supplementary material, which is available to authorized users.

## Background

Respiratory syncytial virus (RSV) is a major cause of respiratory tract infections in young children that frequently leads to hospitalization [1]. The clinical symptoms vary from upper respiratory tract infection to severe bronchiolitis with respiratory insufficiency for which mechanical ventilation is needed. Despite the high disease burden of RSV in young children, no antivirals, vaccines, or other targeted treatments for bronchiolitis have proven to be beneficial yet, and therefore, only supportive care is currently recommended [1–3]. Prematurity, young age (< 6 months), and the presence of siblings or daycare attendance are important risk factors for severe RSV disease requiring hospitalization and respiratory support, indicating that development of the immune system as well as environmental factors plays a role in the pathogenesis of RSV bronchiolitis [4–6].

To date, it is thought that the host response to the virus contributes most prominently to the key features of bronchiolitis, marked by swelling of the mucosa, secretion of mucus, and eventually obstruction of the smaller airways [6–9]. In addition, high viral load in the lungs and nasopharynx have been found in children with severe infection, suggesting that viral replication also contributes to increased pathology [10–12]. During the inflammatory response induced by the virus, cytokines and chemokines are released and attract circulating leukocytes to the site of infection. As recently reviewed by Russel et al., CXCL-8 and CXL-10 have been associated with immune pathology, whereas CCL-5 in general is associated with a more beneficial immune response [7]. It seems that a balanced inflammatory response with influx of immune cells is crucial, as indicated by near absence of cytotoxic CD8 cells in fatal cases of RSV bronchiolitis and the presence of massive influx of neutrophils in the lumen of the airways of most severe infections [13, 14]. The importance of the inflammatory response in the development of severe disease is further underlined by the association of the level of circulating immune cells and secreted cytokines with disease severity [15].

Respiratory mucosal surfaces are colonized directly after birth with bacteria from the mother, and the composition changes dramatically during the first years of life depending on genetics and environmental factors [16–18]. These commensal bacteria release components such as lipopolysaccharides and peptidoglycans that have been shown to pass the epithelial barrier of the mucosal lining under non-inflammatory conditions [19]. This translocation of bacterial products increases during inflammation [20, 21]. Induction of the initial innate immune response in epithelial cells and immune cells by RSV and the secretion of cytokines and chemokines should therefore be considered in the presence of these bacterial products [22].

Findings from the MARC-35 bronchiolitis cohort by Mansbach and coworkers, a prospective study of 1016 infants below 1 year of age hospitalized with bronchiolitis, underpin the importance of bacterial colonization of the respiratory tract during viral infection [23]. They show that depending on infection with RSV and rhinovirus, different compositions of nasopharyngeal microbiota are found, mainly higher levels of *Streptococcus* and *Haemophilus*/*Moraxella,* respectively. Steenhuijsen Piters and co-workers recently showed that clusters characterized by the dominance of *Haemophilus* (*influenzae*) and *Streptococcus* were positively associated with RSV infection and RSV-related hospitalization, based on nasopharyngeal microbiota clusters that were stratified prior to analyses [24]. In addition, they reported enhanced expression of host genes linked to inflammation and immune signaling in patients with the two microbiota clusters (*Haemophilus* and *Streptococcus* dominated), suggesting that interactions between RSV and specific components of the nasopharyngeal microbiota modulate the host immune response, potentially driving clinical disease severity. These findings are further supported by the association of specific metabolic profiles in respiratory samples with severe bronchiolitis, which could be linked to the composition of the microbiome [25]. In this study, we investigate the association of the nasopharyngeal microbiota, without prior stratification on microbiota types, to RSV load, nasopharyngeal cytokine responses, and the association with disease severity.

## Results

### RSV study cohort and baseline characteristics.

**Fig. 1:**
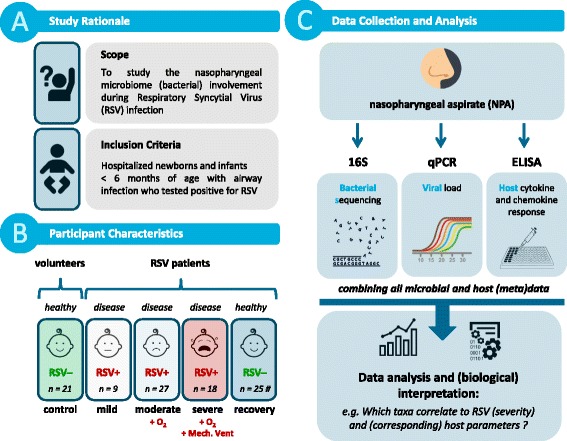
Study design. Graphical summary of the study focus, design, and analysis, as adopted in this manuscript

We retrospectively selected a cohort of 54 infant patients younger than 6 months of age, who were hospitalized with an RSV infection, and 21 age-matched healthy infants (Fig. 1a, Table 1, and Additional file 1: Table S1A). Patients were stratified based on severity of RSV disease using clinically defined parameters as follows: mild disease included children without hypoxemia (*n* = 9); moderate disease included children receiving supplemental oxygen (*n* = 27); and severe disease included children requiring mechanical ventilation (*n* = 18) (Fig. 1b). We collected nasopharyngeal aspirate (NPA) samples from a subset of RSV patients (*n* = 25) 4–6 weeks after hospital discharge, enabling us to evaluate mucosal immune responses and the microbiome after recovery of disease (*n* = 2, 16, and 7, for mild, moderate, and severe, respectively). NPA samples were collected in which bacterial composition, viral load, and host immune responses were measured to allow for integrated analysis (Fig. 1c).

**Table 1:** Descriptive characteristics of the study samples (*n* = 75)

No. of participants (*n* = 75)	Control		Mild		Moderate		Severe		Recovery	
	*n = 21*		*n = 9*		*n = 27*		*n = 18*		*n = 25*	
Age (days)	89	(37)	88	(29)	75	(50)	37	(21)	109	(48)
Hospital Stay (days)	0	(0)	3	(3)	8	(5)	14	(6)	0	(0)
RSV viral load (Ct)	50.0	(0)	22.4	(3.3)	27.0	(5.4)	23.6	(3.4)	50.0	(0)
Weight (gram)	N/A	N/A	14,674	(21111)	5516	(2002)	3874	(931)	5375	(2333)
Birth weight (gram)	2624	(768)	3323	(618)	3271	(656)	2867	(738)	3116	(677)
% antibiotics	0%		22%		7%		11%		8%	
% breastfed	38%		67%		56%		72%		76%	
% males	24%		56%		52%		44%		44%	
Mean number of reads	27,449	(4464)	24,916	(4826)	21,787	(5776)	28,866	(32974)	22,658	(5208)
Total number of reads	5.76E+05		2.24E+05		5.88E+05		5.20E+05		5.66E+05	
Assigned at family	99.72%	(0.01%)	99.88%	(0.01%)	99.90%	(0.01%)	99.94%	(0.01%)	99.91%	(0.01%)
Assigned at genus	94.72%	(0.07%)	97.91%	(0.03%)	98.98%	(0.02%)	98.15%	(0.03%)	97.89%	(0.04%)
Assigned at species^a^	21.22%	(0.19%)	8.07%	(0.06%)	10.25%	(0.09%)	10.27%	(0.09%)	8.43%	(0.17%)
Mean number of OTUs	203	(56)	167	(44)	139	(38)	150	(38)	160	(50)
Total number of OTUs	4270		1499		3756		2705		3999	
Mean Chao index^b^	176.8	(54.4)	151.7	(42.6)	131.1	(39.4)	137.5	(17.8)	150.2	(53.5)
Mean Shannon index^b^	3.1	(1.1)	2.8	(0.7)	2.4	(0.8)	2.7	(0.7)	2.4	(0.8)
Mean PDWT index^b^	6.0	(1.7)	5.6	(1.1)	5.1	(1.6)	4.9	(0.9)	6.1	(2.0)

Data are presented as average + standard deviation (SD) for continuous variables and as percentages for categorical variables. Numbers listed in brackets: SD. The antibiotic use was at the time of infection, and breastfeeding duration was not specified

^a^Note that due to technical limitations in the resolution of 16S marker gene sequencing, OTU (Operational Taxonomic Unit) calling on the level of species should be interpreted with caution

^b^Alpha diversity metrics

To account for potential confounding variables, we screened our cohort on various parameters such as gender, age, (birth) weight, and RSV viral load (Table 1 and Additional file 2: Figure S1). Infant age (and gender to a minor extent) was found to be unevenly distributed over our cohort, which is likely a consequence of age being a known risk factor for developing (severe) RSV infection [26]. Birth weight, although not significantly different between study groups, did show a trend towards being increased in RSV patients in comparison to healthy infants. Antibiotic use was evenly distributed over the RSV patient severity stratifications (Table 1); none of the healthy infants used antibiotics at time of enrollment. Altogether, this prompted us to correct for age, gender, and birth weight effects in multivariate analyses throughout this study. We also assessed viral co-infections, for which we found that only coronavirus (11%) and rhinovirus (20%) had a high prevalence. However, patients with these co-infections appeared to be randomly distributed in the disease severity stratifications (Additional file 1: Table S1B).

### Nasopharyngeal microbiota composition

**Fig. 2:**
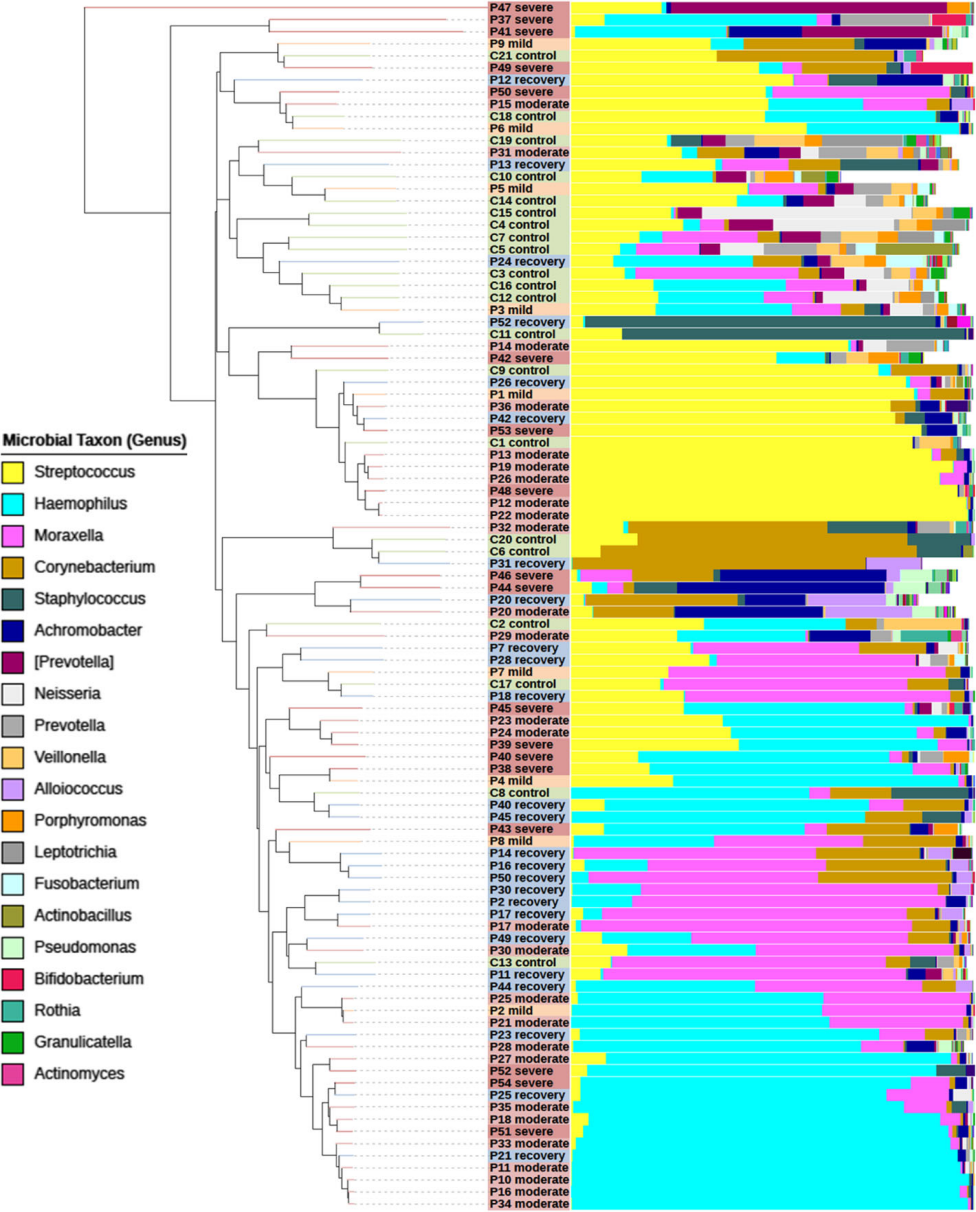
Nasopharyngeal microbiota composition in healthy and RSV-infected infants. Each leaf of the tree represents a single sample. Samples were clustered based on beta diversity (‘between-sample distance’), using weighted UniFrac as a distance measure and hierarchical UPGMA as a clustering method. Vertical bars show the relative abundance microbiota composition on the genus level (reads that could not be classified up to this level are in white). The 20 most dominant genera are shown in the legend. Colored sample labels represent sample classes: mild (orange), moderate (light red), or severe (dark red) disease; healthy control (green); and recovery (blue) samples. The figure was generated with the interactive tree of life (iTOL) program

16S rRNA marker gene Illumina sequencing (16S) of the V3-V4 region was used to study the consortia of bacteria present in the NPA samples. By adopting a customized QIIME-based workflow for analysis of sequencing reads, we were able to classify 97.6% of sequencing reads with confidence to the genus-level and study-wide identified a total of 156 unique genera (Additional file 3: Table S2 and Additional file 4: Table S3). Sufficient sequencing reads and OTU (operational taxonomic unit) counts for each sample were realized with an average of 25k ± 15k SD reads and 162 ± 51 SD OTUs, respectively (Table 1 and Additional file 3: Table S2). We identified *Haemophilus* (30.5% relative abundance on average; healthy and RSV-infected individuals combined), *Streptococcus* (29.4%), *Moraxella* (10.7%), *Corynebacterium* (6.89%), and *Staphylococcus* (2.9%) as the main genera (and known to be typically present) in the nasopharynx (Fig. 2) [24]. In addition, we found minor levels of *Prevotella* (4.2%), *Achromobacter* (3.6%), *Neisseria* (2.2%), and *Veillonella* (1.4%) representing additional genera (Fig. 2).

### RSV disease, viral load, and CXCL8 levels can be explained by nasopharyngeal microbial make-up.

**Fig. 3:**
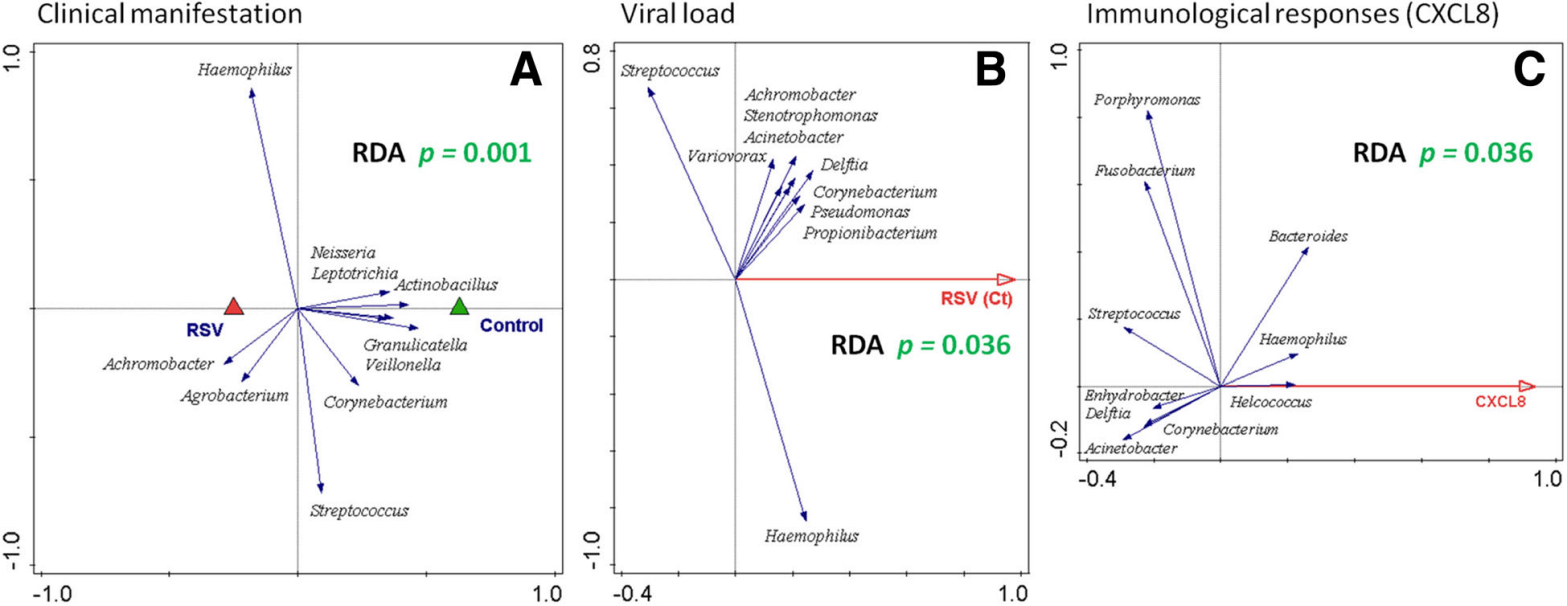
RSV disease, viral load, and CXCL8 levels in healthy and RSV-infected individuals can be explained by nasopharyngeal microbial makeup (genus-level). Redundancy analysis (RDA) biplots are shown. Nasopharyngeal genus-level microbiota from healthy and RSV-infected individuals are significantly different, irrespective of disease severity (**a**) (according to a permutation test; *p* value = 0.001). Triangles are the centroids of the study sample groups: RSV (red) and healthy control (green). RDA of RSV-infected individuals (healthy and recovery samples were excluded from analysis) shows that nasopharyngeal genus-level microbiota can significantly be separated based on viral load (**b**) (Ct threshold plotted: higher Ct number corresponds to higher number of PCR cycles before confident virus detection, hence lower viral load; *p* value = 0.036). For **a** and **b**, the blue arrows are the 10 best-fitting genera (names in italic), which are the genera best explaining microbiota compositional differences between disease status (**a**) or different levels of RSV virus (**b**) as plotted on the horizontal axis. RDA of healthy and RSV-infected individuals shows that nasopharyngeal genus-level microbiota can significantly be separated based on levels of CXCL8 (**c**) (*p* value = 0.036; log transformation was set to 1000). The first component (horizontal axis) is optimized to explain CXCL8 level based on microbiota relative abundances (concentration of CXCL8 in pg/μl). Correspondingly, the blue arrows are the genera (names in italic) explaining at least 5% of this variation. RDA was corrected for age, gender, and birth weight. See Additional file 2: Figure S2 for similar analysis on the OTU level

To examine the microbial involvement in RSV disease processes, redundancy analysis (RDA) was performed and showed a strong and significant separation of RSV patients and healthy controls based on NPA microbial makeup (Fig. 3a; *p* = 0.001). This difference in contrast is mainly characterized by *Haemophilus* and *Achromobacter*, not by *Agrobacterium*, as this bacterium is only represented by three RSV samples with < 0.01% relative abundance each. Both viral load and host immune responses are hypothesized to play a major role in the disease processes, through which microbiota could potentially (directly or indirectly) assert their effect on the pathophysiology and the course of disease. Therefore, we further focused on differences in microbiota content between different viral loads and host cytokines and chemokines that were previously described to be relevant in RSV disease (CCL5, CXCL8/IL8, CXCL10, IL6, and MMP9) [15, 27, 28].

We found that RSV viral load (as determined with qPCR, Ct) in nasopharyngeal aspirates of patients was associated significantly with total nasopharyngeal microbiota composition (Fig. 3b; RDA *p =* 0.036), although no statistically different viral loads between the patient RSV severity stratifications could be detected (Additional file 1: Table S1A). However, because the response variables of the data in the RDA are also strongly oriented in the vertical direction, this indicates that separation based on genus-level microbiota is not primarily driven by the viral load contrast.

Furthermore, RDA of healthy and RSV-infected individuals showed that nasopharyngeal genus-level microbiota can significantly be separated based on levels of CXCL8 (Fig. 3c; RDA *p* = 0.036) but not for any of the other candidate markers (data not shown). Strikingly, in this RDA, *Haemophilus* is among the most important genera contributing to the observed separation, as its arrow points to CXCL8 in a strong horizontal direction. *Helcococcus*, although its arrow is also strongly horizontally oriented, has a very low abundance (0.01% on average).

The above-described genus-level RDA analyses (Fig. 3) were repeated with higher resolution OTU-level microbiota data (clustering on 97.0%), and on this level of taxonomical detail, similar statistical significances and microbial associations were observed (Additional file 2: Figure S2). Mainly, OTUs classified to genus or species of *Haemophilus* were found to be important in driving these outcomes and *Streptococcus* to a lesser extent. However, we did lose significance for the association of CXCL8 with microbiota on the level of OTU in comparison to genus (*p = 0.036* to *p* = 0.089; Additional file 2: Figure S2C), which might in part be governed by birth weight, as removal of birth weight as confounding factor resulted in restoring of statistical significance as observed at the level of genus (*p* = 0.02; data not shown). Interestingly, OTU-level analysis of microbiota associated with RSV viral load provided increased insight with regard to *Haemophilus*-classified OTUs, as these best explained association with viral load but not other OTUs in the top 10 response variables (Additional file 2: Figure S2B). Furthermore, the above-described association of *Helcococcus* with levels of CXCL8 was not observed on the level of OTU (Additional file 2: Figure S2C) and, instead, was likewise best explained by OTUs classified as *Haemophilus* taxa. In conclusion, *Haemophilus* seems to be a strong correlate to the outcome for clinical manifestation of RSV disease, viral load, and CXCL8 immune response.

### RSV disease is marked with increased populations of *Haemophilus* and *Achromobacter*

**Fig. 4:**
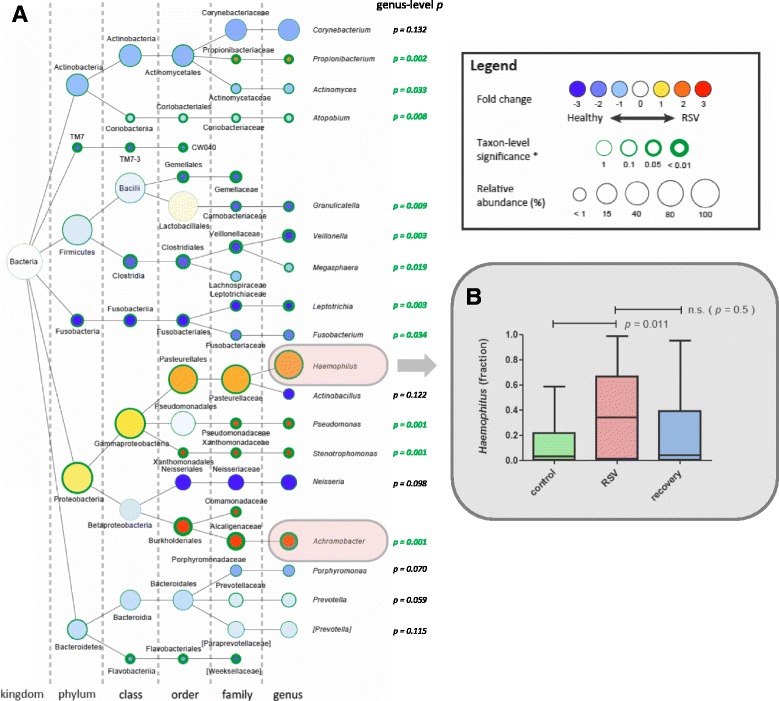
Difference in nasopharyngeal microbial community composition between healthy and RSV-infected individuals is strongly characterized by an overrepresentation of *Haemophilus*. The strongest differentially abundant microbial taxa for healthy versus RSV-infected individuals are shown in a graphical Cytoscape visualization (**a**) [51]. Nodes represent taxa (node size represents average relative abundance (i.e., dominance) for both experimental groups combined); edges (dashed lines) link the different taxonomic levels. The weighed fold-change (node color) is calculated as the ^2^log of the ratio of the relative abundance between healthy and RSV (0 = no difference between disease state, 1 = twice as abundant in RSV, etc.). So, yellow to red indicates an overrepresentation during RSV infection, hence an underrepresentation in healthy infants and vice versa for light to dark blue. The significance (node border width) is expressed as the *p* value of a Mann–Whitney *U* test, FDR-corrected for multiple testing. The genus-level *p* values are listed on the right of the genera nodes. We observe a strong and significant overrepresentation of *Haemophilus* genus during RSV infection (*p =* 0.011) (**b**) and of *Achromobacter* (*p =* 0.001) (Additional file 2: Figure S4A). For recovery versus RSV samples, significance was determined using Wilcoxon signed rank test

Further evaluation of microbial nasopharyngeal (alpha) diversity between study groups suggested that RSV infection is characterized by decreased species richness (*p =* 0.006; MWU, corrected), which is more pronounced in moderate and severe disease compared to controls (*p* = 0.016 and *p* = 0.024) (Additional file 2: Figures S3A and S3C). Regarding species diversity, no significant differences associated with RSV-infected infants or severity of disease were found (Additional file 2: Figures S3B and S3D). A trend in reduction of species diversity in RSV patients was observed here (*p* = 0.098), and this species diversity, interestingly, is partly restored after disease recovery (*p* = 0.060, uncorrected; Additional file 2: Figure S3B). Furthermore, microbial makeup of the nasopharynx of RSV-infected compared to healthy individuals was characterized by a strong and significant overrepresentation of *Haemophilus* (Fig. 4), increasing from 11.8 to 37.8% relative abundance (*p =* 0.011; MWU, corrected) and (with lower abundance) of *Achromobacter* (Additional file 2: Figure S4A; increasing from 0.7 to 4.7%; *p =* 0.001). After recovery from RSV disease, based on a paired sample analysis, the level of *Haemophilus* was restored in these infants and was found to be similar to the level of healthy volunteers (Fig. 4b). In contrast, a significant underrepresentation of *Veillonella* and *Leptotrichia* was observed during RSV infection (Additional file 2: Figures S4B and S4C), which however might be a consequence of the increase of the aforementioned bacteria. On a side note, *Moraxella* appeared to be primarily present in recovery samples from RSV-infected individuals (Fig. 2 and Additional file 2: Figure S5). In conclusion, RSV infection caused a strong microbial perturbation characterized by presence of *Haemophilus* and *Achromobacter*.

### Mucosal IL6 and CXCL8 responses correlate with clinical RSV disease severity but not with a specific microbiota profile

**Fig. 5:**
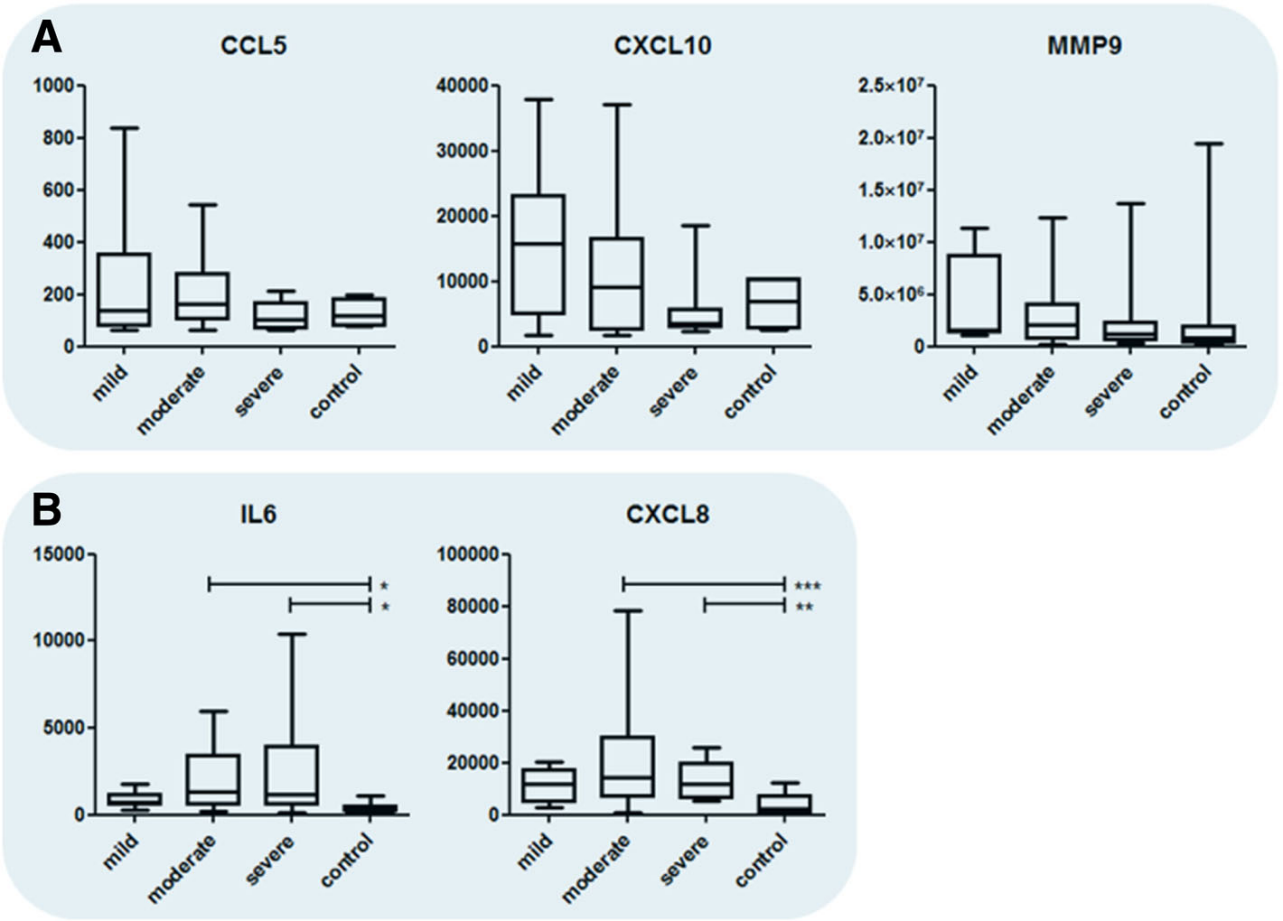
Chemokine and cytokine levels during RSV infection. Chemokine and cytokine levels in nasopharyngeal aspirates of healthy, RSV-infected individuals with different disease severities (**a**, **b**). In general, host responses are observed to be negatively (**a**) or positively (**b**) correlated with RSV severity. Data is presented in pg/ml. Statistics in these plots were obtained by Kruskal–Wallis one-way ANOVA, with Dunn’s correction for multiple testing. Significances are as follows: *p < 0.05, **p < 0.01, ***p < 0.001

Local host response measurements in the NPA indicated that cytokines and chemokines are either negatively (CCL5, CXCL10, and MMP9; Fig. 5a) or positively (CXCL8 and IL6; Fig. 5b) correlating with RSV disease severity. This was statistically confirmed for IL6 and CXCL8 by Spearman correlation (*p =* 0.0009/rho = 0.39 and *p =* 0.0001/rho = 0.51, respectively; uncorrected), although between RSV sample groups only, we did not find significant differences with ANOVA. As severity measure based on oxygen and mechanical ventilation requirement is a non-continuous value, we validated the above numbers by correlating with duration of hospital stay, which is an accepted measure of RSV severity, and found similar correlations (*p =* 0.0005/rho = 0.41 and *p =* 0.0003/rho = 0.46, for IL6 and CXCL8, respectively; uncorrected). Furthermore, paired analysis with recovery samples indicated that the IL6 and CXCL8 cytokine responses were restored to normal levels after recovery from RSV disease, as the responses after recovery were significantly lower than for their corresponding sample upon disease (IL6 *p* = 0.02; CXCL8 *p* = 0.005; Additional file 2: Figure S6).

**Fig. 6:**
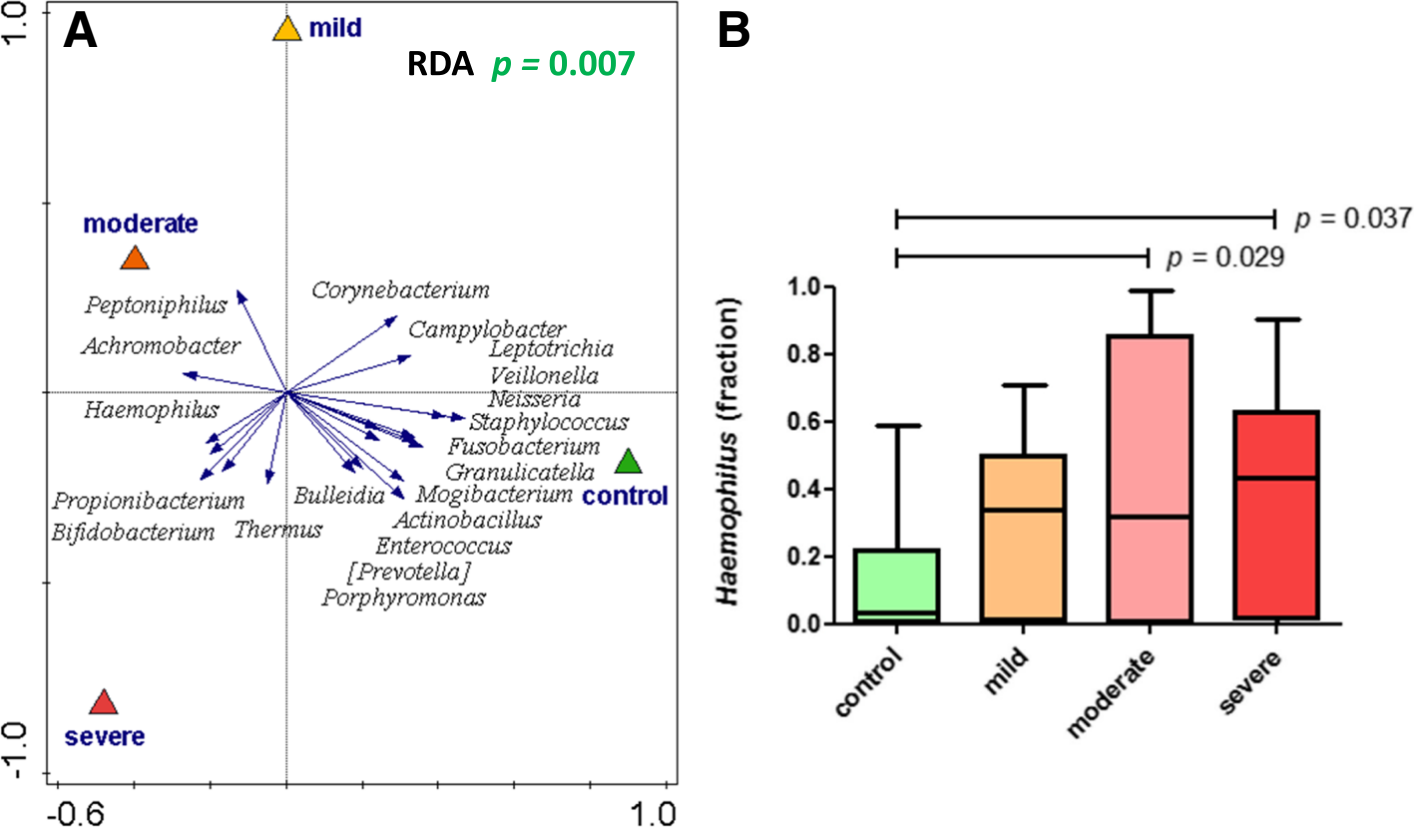
Nasopharyngeal microbiota composition allows for separation of healthy and RSV-infected individuals. Nasopharyngeal genus-level microbiota from healthy and RSV-infected individuals are significantly different (**a**). The figure shows a redundancy analysis (RDA) biplot. Triangles are the centroids of the study sample groups: mild (yellow), moderate (orange), and severe (red) RSV and healthy control (green). The blue arrows are the 20 best-fitting bacterial genera (names in italic), i.e., taxa that best explain the differences between the sample groups. The horizontal axis maximizes the variation in sample groups (in contrast to a principal component analysis plot, where the variation between individual samples is maximized). In RDA, samples (also) separated in the vertical direction indicate that this separation is (also) driven by other factors than the primary contrast, such as by individuality. The difference in microbiota is significant (according to a permutation test; *p =* 0.007). We observe a strong and significant overrepresentation of *Haemophilus* genus in RSV (*p =* 0.011; MWU, FDR-corrected) especially in moderate and severe RSV infections (**b**) and of *Achromobacter* (*p =* 0.001) (Additional file 2: Figure S4A)

In line with our findings as described above, RDA indicated that healthy infants and RSV-infected patients do indeed display very distinct microbial patterns (Fig. 6a; RDA *p* = 0.007). However, this RDA separation seemed to be primarily driven by disease state rather than disease severity, as an additional RDA on the subset of RSV patients alone did not yield convincing nor significant separation of RSV severity groups (data not shown; RDA *p* = 0.75). Correspondingly, levels of *Haemophilus* did not differ significantly between patient groups with distinct disease severities (Fig. 6b); although in comparison to control subjects, *Haemophilus* levels were higher in moderate and severe patients than in mild.

Interestingly, based on an extended in silico analysis of the dominant *Haemophilus*-level-classified OTUs detected in this study (> 0.01% average relative abundance, *n* = 10), we conclude that *Haemophilus* data described in this study predominantly belongs to the species of *Haemophilus influenza* (Additional file 2: Figure S7)*.* The top 10 most abundant *Haemophilus*-classified OTUs together comprise 99.65% of the reads belonging to all 361 detected *Haemophilus* genus OTUs (Additional file 5: Table S4). Of these 10 OTUs, one could confidently be assigned to *H*. *parainfluenzae* and comprise 0.25% of all reads in this study, and two other OTUs were assigned to an unknown cluster (0.31%). The remaining seven OTUs, the major fraction of *Haemophilus*-assigned reads, could confidently be assigned to *H*. *influenza* (27.7% of all reads in this study). Other candidate taxa involved in the RDA separation, such as *Achromobacter*, *Veillonella*, and *Leptotrichia* were not highly abundant in the overall microbiome and did not correlate with disease severity (Additional file 2: Figure S4). In conclusion, RSV is marked by a strong increase in *Haemophilus* and to lesser extent *Achromobacter*, but RSV disease severity cannot be related to microbiota composition.

## Discussion

Understanding why some children develop severe bronchiolitis while most children experience an upper respiratory tract infection upon RSV infection remains essential and needs to be answered to improve the care of RSV-infected children in the future. Where several previous studies focused on the microbial content involved in bronchiolitis, most notably by Hasegawa and coworkers, our study exclusively focused on RSV-implicated bronchiolitis [29, 30]. RSV disease severity is a multifactorial problem, in which the viral load and the inflammatory response are important drivers of disease, although this is mainly true in previously healthy children whose airways are normal [6, 7, 12]. An important question this study tried to answer is whether nasopharyngeal microbiome composition relates to local viral load and exerts an influence on mucosal immune responses. Viral load and mucosal immune responses are thought to directly impact disease severity, and therefore, it is difficult to disentangle these direct and indirect effects of the microbiome on disease outcome. The alpha diversity of the microbiota from patients with an acute RSV infection showed a decrease in species richness but not in diversity compared to healthy infants. This means that the number of different species reduces while there is no difference in the distribution of the numbers of each species. A reduced alpha diversity of the upper respiratory tract has also been shown in a longitudinal cohort study of symptomatic rhinovirus infections in infants (< 1 year) [31] and after administration of an intranasal live-attenuated influenza vaccine in adults [32]. Both studies were performed prospectively and show that lower alpha diversity is caused by viral infection and is not a prerequisite for viral infection. In another observational study, comparing the microbiome during RSV and rhinovirus infections, it was shown that there might be an association with different nasopharyngeal microbiome profiles, although this has not been confirmed by others yet [33]. This suggests that the type of alteration of the microbiome might be specific for RSV infection but also could be a more general effect of immune system perturbation. Yet, it is uncertain whether the observed microbiome changes in our study are the cause or effect of RSV infection.

A multivariate redundancy analysis (RDA) could separate healthy controls from RSV-infected infants but could not separate microbiomes based on the level of disease severity. In the study of Steenhuijsen Piters et al., the authors concluded that the microbiota composition might affect the clinical severity, but we were unable to reproduce these findings, perhaps because different RSV severity measures were applied. Nevertheless, a strong and significant overrepresentation of *Haemophilus* was found in RSV-infected children, especially in severe and moderate disease. In a recent study, Rosas-Salazar and coworkers observed a similar increase of *Haemophilus* in infants with acute RSV infection compared to healthy controls and also of *Streptococcus* and *Moraxella* [34]. In line with these and our own findings, Hasegawa and coworkers reported that infants with RSV infection and *Haemophilus*-dominant profiles had higher odds of intensive care use than RSV-infected infants with profiles dominated by other bacteria, such as *Moraxella* [30]. In the MARC-35 bronchiolitis cohort by Mansbach and coworkers, lower levels of *Haemophilus*/*Moraxella* were found in the nasopharynx of RSV-infected infants in comparison to rhinovirus; however, this study had no data on healthy infants [23]. Although in our studies *Achromobacter* shows a much lower abundance than *Haemophilus,* it appeared to be also dominant in RSV-infected infants. Apart from a few studies describing the association of *Achromobacter xyloxosidans* with pneumonia in newborns and young infants, and chronic infection in cystic fibrosis patients, not much is known about this species [35–37]. Much more in vitro and in vivo evidence is present with regard to increased susceptibility for viral infections and increased inflammatory responses associated with *Haemophilus* colonization and infection [38–42]. However, to the best of our knowledge, this is the first time that it is shown that the composition of the microbial colonization in the nasopharynx is associated with a higher RSV load, with *Haemophilus* (*influenzae*) as most prominent candidate contributing to these differences (Additional file 2: Figure S2B). Interaction of *Haemophilus* with epithelial cells may lead to the suppression of anti-viral immune pathways allowing increased replication. An alternative explanation would be that RSV facilitates the colonization of *Haemophilus*. However, no evidence for any of the two hypotheses can be found in the current literature. The *Helcococcus* genus*,* another lead based on our reported association with levels of CXCL8 in the NPA (Fig. 3c), is not likely to have a great impact on its niche, as it is very lowly abundant (0.01% on average), and is only represented by three RSV patients, and the association was not observed at the level of OTUs. The increase in IL6 and CXCL-8 responses in RSV patients colonized with *Haemophilus* as found in our study corroborates a recent in vitro study by Gulraiz et al. They showed that release of IL6 and CXCL-8 after RSV infection, but not rhinovirus infection, was synergistically increased in *Haemophilus influenzae* pre-treated human bronchial epithelial cells [40], suggesting that the interaction between *H*. *influenzae*, CXCL8, and RSV may be specific for RSV infections. It should finally be noted that other cytokine responses than the ones studied here might be important for RSV pathogenesis, such as T-helper 1, T-helper 2, and T-regulatory cell type cytokines [43]. Their potential involvement with the nasopharyngeal microbiome during RSV pathogenesis can therefore not be confirmed within the current study.

Interestingly, Steenhuijsen Piters et al. recently reported an enhanced (non-significant) CXCL8 gene expression response, measured in a whole blood transcriptome analysis, that was associated with nasopharyngeal microbiomes that were dominated by *Haemophilus* [24]. In our study, we are able to show a significant association between nasopharyngeal CXCL8 levels and RSV infection and even with disease severity. Although presence of *Haemophilus* was related to the levels of CXCL8, we could not confirm a direct relation between the composition of the microbiome and disease severity. A possible explanation could be that although we stratified the patients on severity level, all patients included in our study were hospitalized and therefore severely ill. The differences in disease severity might have been too subtle to correlate them to microbial composition. In contrast to the study from Steenhuijsen Piters et al., we chose to follow a more unbiased approach and therefore we did not stratify for ‘nasotypes’ on forehand. This may have led to the fact that we could not confirm a relation between *Streptococcus* and disease severity. In a previous study, we even found a reversed relation between *Streptococcus pneumoniae* and disease severity, although this was based on qPCR data and not on 16S sequencing [25, 44, 45]. It should also be noted that although we rigorously corrected our data analyses for a number of potential confounders (i.e., age, gender, and birth weight), we did not have socioeconomic information of our cohort, nor on mode-of-delivery, which can be seen as limitation of this study.

In conclusion, although no association of the nasopharyngeal microbiota to disease severity was found, we show that RSV infection affects the microbiota composition. *Haemophilus*-dominated profiles were in part associated with an increased viral load and increased IL6 and CXCL-8 responses on the genus level of, and these effects were even stronger on the level of OTU (*Haemophilus influenzae*). Upon recovery, *Moraxella* appears to thrive in the previously RSV-perturbed microbiomes. A better understanding of the mechanisms behind the influence of the microbiota on these host-virus processes is needed and should be the focus of future research. Improved insight in microbiome effects on RSV pathogenesis might pave the way for new preventive and therapeutic strategies to reduce the burden by RSV disease.

## Conclusions

Severity of RSV infection in infants is determined by several factors. A growing body of evidence suggests that the nasopharyngeal microbiome may play an important role and influence both the local immune response as well as viral load. Interactions between RSV, local mucosal nasopharyngeal microbial content, and the plethora of host factors involved are not well enough understood to explain severity of disease. This retrospective study using a well-characterized cohort of young children with RSV infection and age-matched healthy controls shows for the first time that composition of nasopharyngeal microbiota associates with CXCL8 levels in RSV patients. CXCL8 is an important chemokine that correlated with RSV disease severity. *Haemophilus* was identified as the most important genus associating with the amplitude of the CXCL8 response. Host-microbe interactions are increasingly recognized as important factors in determining outcome of host processes. Our findings show a strong interdependency between the nasopharyngeal microbiota and the mucosal immune response, potentially influencing severity of disease. These data contribute to a better understanding of the importance of commensal microbiota in respiratory health and disease.

## Methods

### Study design

This study was performed in two hospitals in Nijmegen, Radboud University Medical Center and Canisius Wilhelmina Ziekenhuis (CWZ). From the area of Nijmegen, children younger than 2 years of age with laboratory-confirmed RSV infections were prospectively included during three consecutive winter seasons (2010/2011, 2011/2012, and 2012/2013), if they were hospitalized to the pediatric ward or intensive care unit (PICU) [46]. Written informed consent was obtained from all parents. Because the peak incidence of (severe) bronchiolitis is below the age of 6 months, and to limit the variation in age-related effects on microbiome and inflammatory response, we only included the children younger than 6 months with a PCR-confirmed RSV infection. Healthy age-matched controls admitted to the hospital for surgery, who needed an elective inguinal hernia correction and had no signs of respiratory infection and a negative PCR for RSV, were included. We obtained permission from the medical ethical commission of the Radboud University Medical Center to collect control samples during 1 year, resulting in 21 samples as used in this study. Patients younger than 6 months with PCR-confirmed RSV-positive bronchiolitis were selected and divided into three groups. Children without hypoxemia were classified as ‘mildly ill’; ‘moderately ill’ children received supplemental oxygen, while ‘severely ill’ children required mechanical ventilation. Patients with congenital heart or lung disease, immunodeficiency, or glucocorticoid use were excluded. Within 24 h after admission, a nasopharyngeal aspirate (NPA) was collected and parents from hospitalized children were asked for permission to collect a second NPA sample 4–6 weeks after admission (recovery). The final study cohort as reported here existed of *n* = 21 healthy infants and *n* = 9 mild (2), *n* = 27 moderate (16), and *n* = 18 severe (7) patients (recovery samples in brackets). For more details on cohort design, available demographics, and sample characteristics, we refer to Additional file 1: Table S1.

### Nasopharyngeal aspirate collection and diagnostics

The nasopharyngeal aspirates (NPA) were collected by introducing a catheter into the nasopharyngeal cavity. For viral diagnostics, samples were analyzed by multiplex PCR, quantifying 15 different viral pathogens: influenza virus types A and B, coronavirus 229E and OC43, human bocavirus, enterovirus, adenovirus, parechovirus, PIV types 1–4, human metapneumovirus, rhinovirus (RV), and RSV, as previously described [47]. See Additional file 1: Table S1B for co-infection information of the samples. IL-6, CXCL8, CXCL10, CCL5, and MMP9 levels were measured by ELISA as described in Additional file 6: Supplementary Methods.

### 16S rRNA gene amplification prior to sequencing

Bacterial DNA extraction and quantification was performed as previously described [48] with some modifications as reported in Additional file 6: Supplementary Methods. To generate the PCR amplicon libraries, sample-specific barcoded amplicons for the V3-V4 hypervariable region of the small subunit ribosomal RNA 16S genes were generated using a two-step PCR. Ten to twenty-five nanograms genomic (g)DNA was used as template for the first PCR with a total volume of 50 μl using the 341F (5’-CCT ACG GGN GGC WGC AG-3′) and the 785R (5’-GAC TAC HVG GGT ATC TAA TCC-3′) primers appended with Illumina adaptor sequences.

### 16S rRNA marker gene sequencing

Illumina 16S rRNA amplicon libraries were generated and sequenced at BaseClear BV (Leiden, The Netherlands) on an Illumina MiSeq paired-end 300 system. Sequencing data analysis was performed by QIIME and is more elaborately described in Additional file 6: Supplementary Methods. The Ribosomal Database Project [49] classifier version 2.3 was performed for taxonomic classification of the sequence reads. Alpha diversity metrics (PD whole tree, Chao1, Observed Species, and Shannon) were calculated by bootstrapping 4822 reads per sample and taking the average over 10 trials. Figures resulting from QIIME clustering analyses were generated using the interactive tree of life (iTOL) tool [50]. For visualization of the differential microbiome, Cytoscape software version 3.1.3 was used [51].

### Statistics

For the microbiota data, statistical significance between contrasts with regard to taxonomy abundances was tested by a non-parametric (unpaired) Mann–Whitney *U* (MWU) test, corrected with false discovery rate (FDR) for multiple testing; unless stated otherwise. Multivariate redundancy analysis (RDA) and principal component analysis (PCA) was done using Canoco 5.04 [52]. For all other experimental data (i.e., protein measurements, metadata, etc.), statistical significance was tested likewise using a non-parametric Kruskal–Wallis one-way ANOVA, with Dunn’s correction for multiple testing (GraphPad Prism 5.0), unless stated otherwise.

For a more detailed description of this materials and methods section, we refer to Additional file 6: Supplementary Methods.

## Additional files

Additional file 1: Table S1. Participant metadata and characteristics. (XLSX 37 kb)

Additional file 2: Figure S1. Potential confounding characteristics of this study. Figure S2 RSV disease and viral load in healthy and RSV-infected individuals can be explained by nasopharyngeal microbial makeup (OTU-level). Figure S3 Species richness, but not diversity, is reduced in RSV-infected infants. Figure S4 Difference in *Achromobacter*, *Veillonella*, and *Leptotrichia* abundance between healthy and RSV-infected individuals with different disease severities. Figure S5 RSV-infected individuals with mild disease symptoms display an ‘intermediate’ nasopharyngeal microbiota compositional profile in comparison to moderate/severe disease and their recovery controls. Figure S6 Chemokine and cytokine levels during RSV infection and upon recovery. Figure S7 Comparison of OTUs from current study to *Haemophilus* reference species shows that *Haemophilus*-classified OTUs are predominantly belonging to *Haemophilus influenzae* species. (DOCX 968 kb)

Additional file 3: Table S2. Sample sequencing data numbers and metrics. (XLSX 25 kb)

Additional file 4: Table S3. Sample microbiota compositional table. (XLSX 247 kb)

Additional file 5: Table S4. Sample OTU table. (XLSX 1629 kb)

Additional file 6: Supplementary information on methods. (DOCX 20 kb)

## Abbreviations

16S: The 16S ribosomal RNA (rRNA) molecule of bacteria and archaea (prokaryotes)
CCL5: (C-C motif) chemokine ligand 5, also RANTES
CXCL10: (C-X-C motif) chemokine ligand 10
CXCL8: (C-X-C motif) chemokine ligand 8, also interleukin-8
ELISA: Enzyme-linked immunosorbent assay
ENA: European Nucleotide Archive
FDR: False discovery rate
gDNA: Genomic DNA
IL6: Interleukin-6
iTOL: Interactive tree of life
MMP9: Matrix metallopeptidase 9
MWU: Mann–Whitney *U* statistical test
NPA: Nasopharyngeal aspirate
OTU: Operational taxonomic unit
PCA: Principal component analysis
PDWT: Phylogenetic distance whole tree
PICU: Pediatric intensive care unit
qPCR: Quantitative PCR
RDA: Redundancy analysis
RSV: Respiratory syncytial virus
RV: Rhinovirus
SD: Standard deviation
UPGMA: Unweighted Pair Group Method with Arithmetic Mean, a hierarchical clustering method
V3-V4: Variable regions V3 and V4 of the 16S rRNA gene
