# Supplementary material for: Haemophilus is overrepresented in the nasopharynx of infants hospitalized with RSV infection and associated with increased viral load and enhanced mucosal CXCL8 responses
Source: Microbiome. 2018 Jan 11;6:10. doi: 10.1186/s40168-017-0395-y (PMC5765694; doi:10.1186/s40168-017-0395-y)
Supplement: Supplementary file 2 — Potential confounding characteristics of this study. Figure S2 RSV disease and viral load in healthy and RSV-infected individuals can be explained by nasopharyngeal microbial makeup (OTU-level). Figure S3 Species richness, but not diversity, is reduced in RSV-infected infants. Figure S4 Difference in Achromobacter, Veillonella, and Leptotrichia abundance between healthy and RSV-infected individuals with different disease severities. Figure S5 RSV-infected individuals with mild disease symptoms display an ‘intermediate’ nasopharyngeal microbiota compositional profile in comparison to moderate/severe disease and their recovery controls. Figure S6 Chemokine and cytokine levels during RSV infection and upon recovery. Figure S7 Comparison of OTUs from current study to Haemophilus reference species shows that Haemophilus-classified OTUs are predominantly belonging to Haemophilus influenzae species. (DOCX 968 kb) [file 40168_2017_395_MOESM2_ESM.docx]

**Supplementary FIGURES**

**Figure S1: Potential confounding characteristics of this study**. A selection of cohort characteristics that potentially could bias the results were evaluated based on RSV disease severity: age in days (**A**), RSV viral load in collected nasopharyngeal aspirate by PCR (**B**) (Ct threshold plotted on y-axis: higher Ct number corresponds to higher number of PCR cycles before confident virus detection; hence lower viral load), weight at birth in gram (**C**), and infant weight at day of hospital intake in gram (**D**). Infant age (and consequently weight) during RSV disease correlates strongly to severity of disease. Therefore, age (but also gender, see Table 1) was consistently corrected for in multivariate statistics throughout this manuscript. Statistics in these plots by Kruskal–Wallis one-way ANOVA, with Dunn's correction for multiple testing. Significances as follows: * *p* < 0.05, ** *p* < 0.01, *** *p* < 0.001. We refer to Table 1, and Additional file 2: Table S1 for further (numeric) details with regard to study sample metadata.


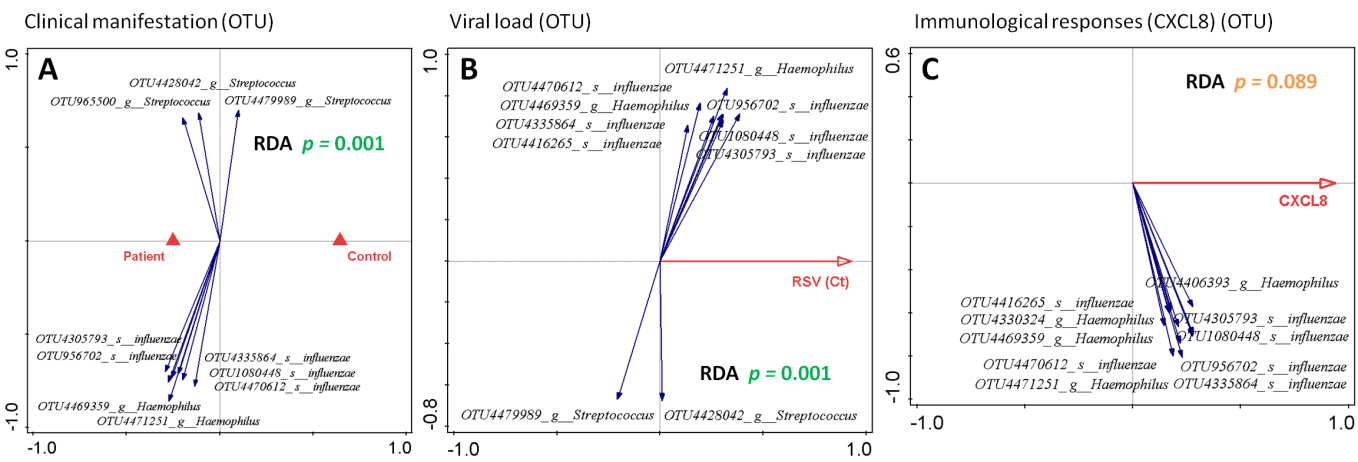


**Figure S2: RSV disease and viral load in healthy and RSV-infected individuals can be explained by nasopharyngeal microbial make-up (OTU-level).** Redundancy analysis (RDA) biplots are shown. Nasopharyngeal OTU-level microbiota from healthy and RSV-infected individuals are significantly different, irrespective of disease severity (**A**) (according to a permutation test; *p-*value = 0.001). Triangles are the centroids of the study sample groups: RSV and healthy control. RDA of RSV-infected individuals shows that nasopharyngeal OTU-level microbiota can significantly be separated based on viral load (**B**) (Ct threshold plotted: higher Ct number corresponds to higher number of PCR cycles before confident virus detection; hence lower viral load; *p-*value = 0.001). For **A** and **B**: The blue arrows are the 10 best-fitting OTU (names in italic; .._g_.. for genus-assigned OTU, .._s_.. for species), i.e. best explaining microbiota compositional differences between disease status (**A**) or different levels of RSV virus (**B**) as plotted on the horizontal axis. RDA of healthy and RSV-infected individuals shows that nasopharyngeal OTU-level microbiota cannot, in contrast to genus-level (*p-*value = 0.036), confidently be separated based on levels of CXCL8 (**C**) (*p-*value = 0.089; log transformation was set to 1000). The first component (horizontal axis) is optimized to explain CXCL8 level based on microbiota relative abundances (concentration of CXCL8 in pg/µl). Correspondingly, the blue arrows are the OTU (names in italic, etc.) explaining at least 5% of this variation. See Fig. 3 in the main text for similar analysis on genus-level. RDA were corrected for age, gender and birth weight.

**
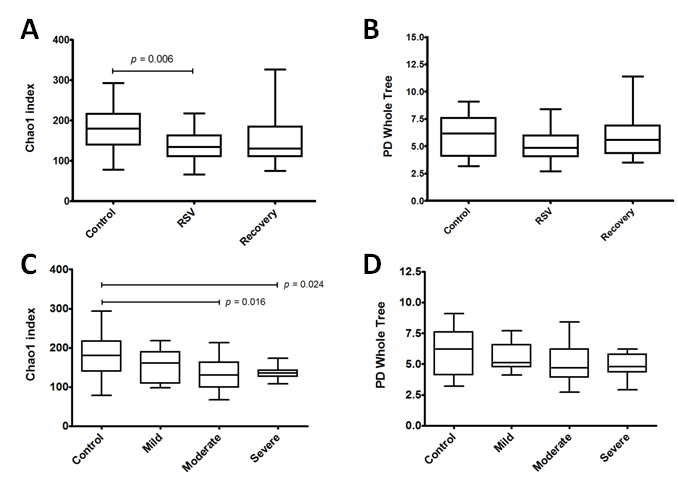
**

**Figure S3: Species richness, but not diversity, is reduced in RSV-infected infants**. Microbial nasopharyngeal richness (Chao1) and phylogenetic diversity (PD) whole tree of healthy versus RSV-infected infants and their respective recovery samples (**A-B**) and of healthy infants versus different severity stratifications of RSV (**C-D**). RSV infection, irrespective of disease severity, in comparison to healthy controls is represented by a decrease in species richness (**A**) but not in species diversity (**B**), as the last does not survive multiple testing. Although borderline significant, it is interesting to observe that this species diversity is partly restored after disease recovery (*p* = 0.060, uncorrected). In a similar fashion, in comparison to healthy volunteers, the microbiota species richness is significantly decreased in moderate and severe RSV (**C**), whereas this is not the case for species diversity as these similar comparisons do not survive multiple testing correction (**D**). Statistics shown in these plots by two-tailed MWU, Bonferroni-corrected for multiple-testing. For recovery versus RSV samples, paired statistics was applied by Wilcoxon signed rank test.

**
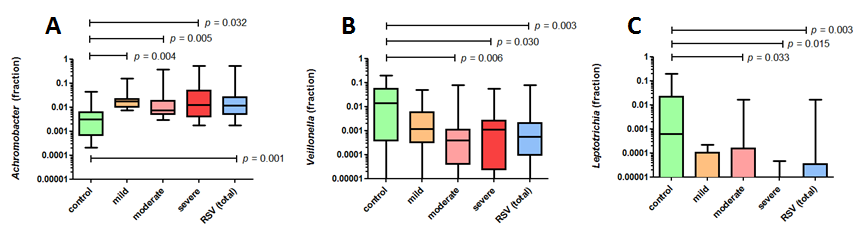
**

**Figure S4: Difference in** ***Achromobacter*, *Veillonella* and *Leptrotrichia* abundance between healthy and RSV-infected individuals with different disease severities.** Shown are box plots of relative abundances (fractions) of *Achromobacter* (**A**), *Veillonella* (**B**) and *Leptrotrichia* (**C**) genera in healthy infants, and mildly, moderately and severely RSV infected infants, and to the total group of RSV patients. The significances of healthy versus total number of RSV individuals (i.e. irrespective of disease severity) are indicated under the graphs. Statistics in these plots by two-tailed MWU, FDR-corrected for multiple testing.

**
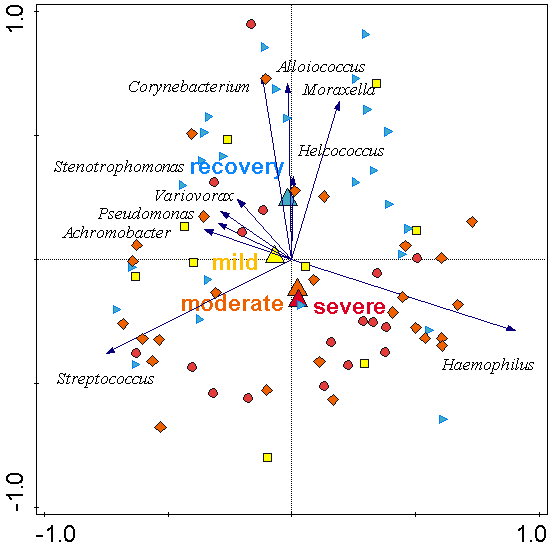
**

**Figure S5: RSV-infected individuals with mild disease symptoms display an “intermediate” nasopharyngeal microbiota compositional profile in comparison to moderate/severe disease and their recovery controls.** Nasopharyngeal genus-level microbial composition in mild, moderate and severely infected RSV individuals, and (for those available) their respective recovery samples. The figure shows a principal component analysis (PCA) plot. Small squares, diamonds and circles represent mild, moderate and severe RSV patients, respectively. Furthermore, small blue triangles represent the recovery samples. Large triangles are the centroids of the study sample groups: mild (yellow), moderate (orange), severe (red) and recovery (blue). The blue arrows are the 10 best-fitting bacterial genera (names in italic), i.e. the taxa that best explain the difference between the sample groups. Interestingly, an RDA on this same contrast (mild, moderate and severe RSV, and recovery) yields a significant sample group separation of *p =* 0.02 (data not shown). PCA was corrected for age, gender and birth weight.


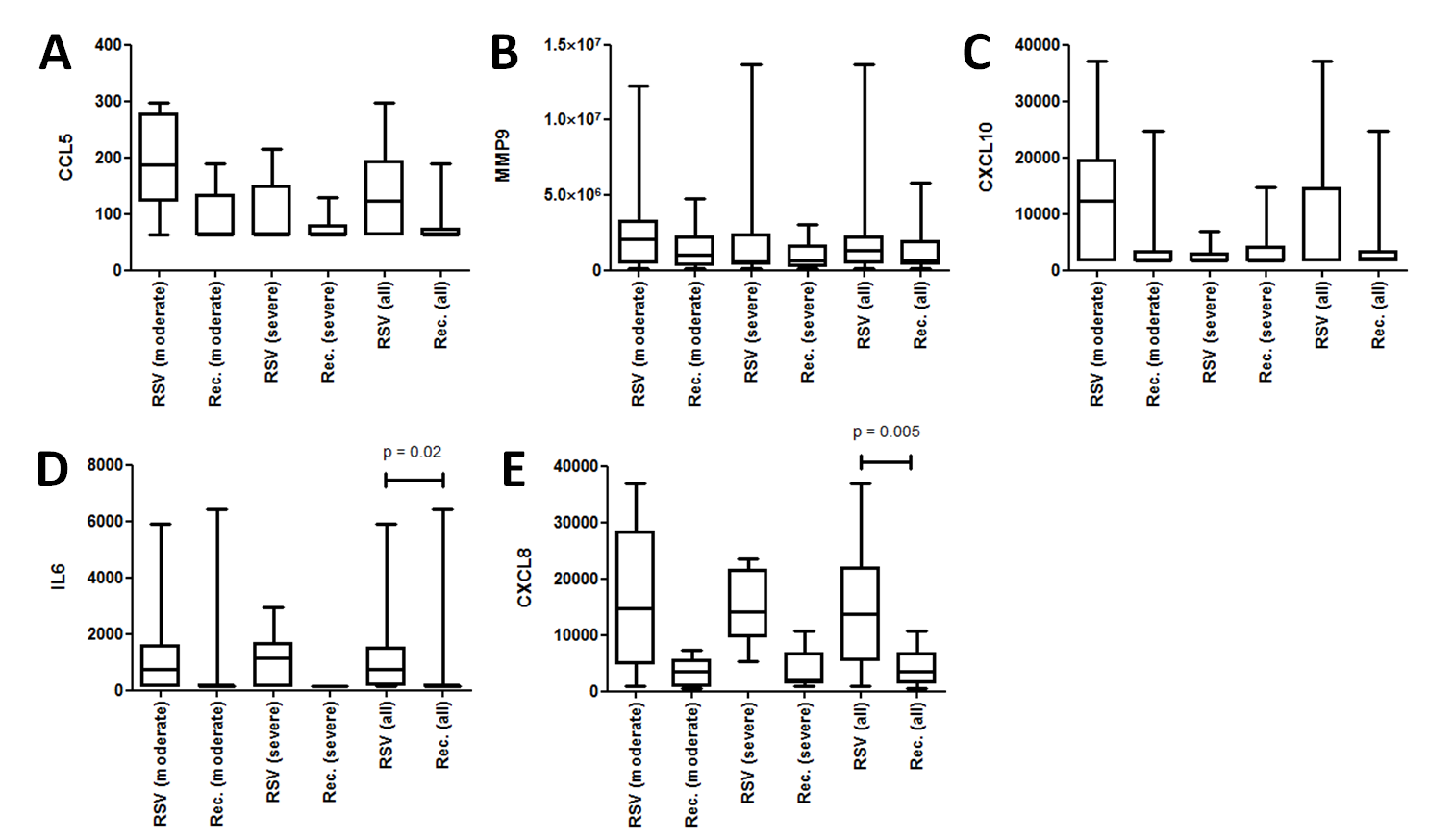


**Figure S6:** **Chemokine and cytokine levels during RSV infection and upon recovery.** Paired analysis of chemokine and cytokine levels in nasopharyngeal aspirates of RSV-infected individuals with moderate, severe or altogether (all) disease severity, and their corresponding recovery samples (Rec.) for CCL5 (**A**), MMP9 (**B**), CXCL10 (**C**), IL6 (**D**) and CXCL8 (**E**). For mild severity, the available number of paired samples was too low for statistical analysis, and therefore not shown (n =2). For moderate and severe n = 6-11 paired samples. Data is presented in pg/ml. Statistics in these plots on the appropriate groups are by paired Wilcoxon signed rank test, Bonferroni-corrected for multiple-testing.

**
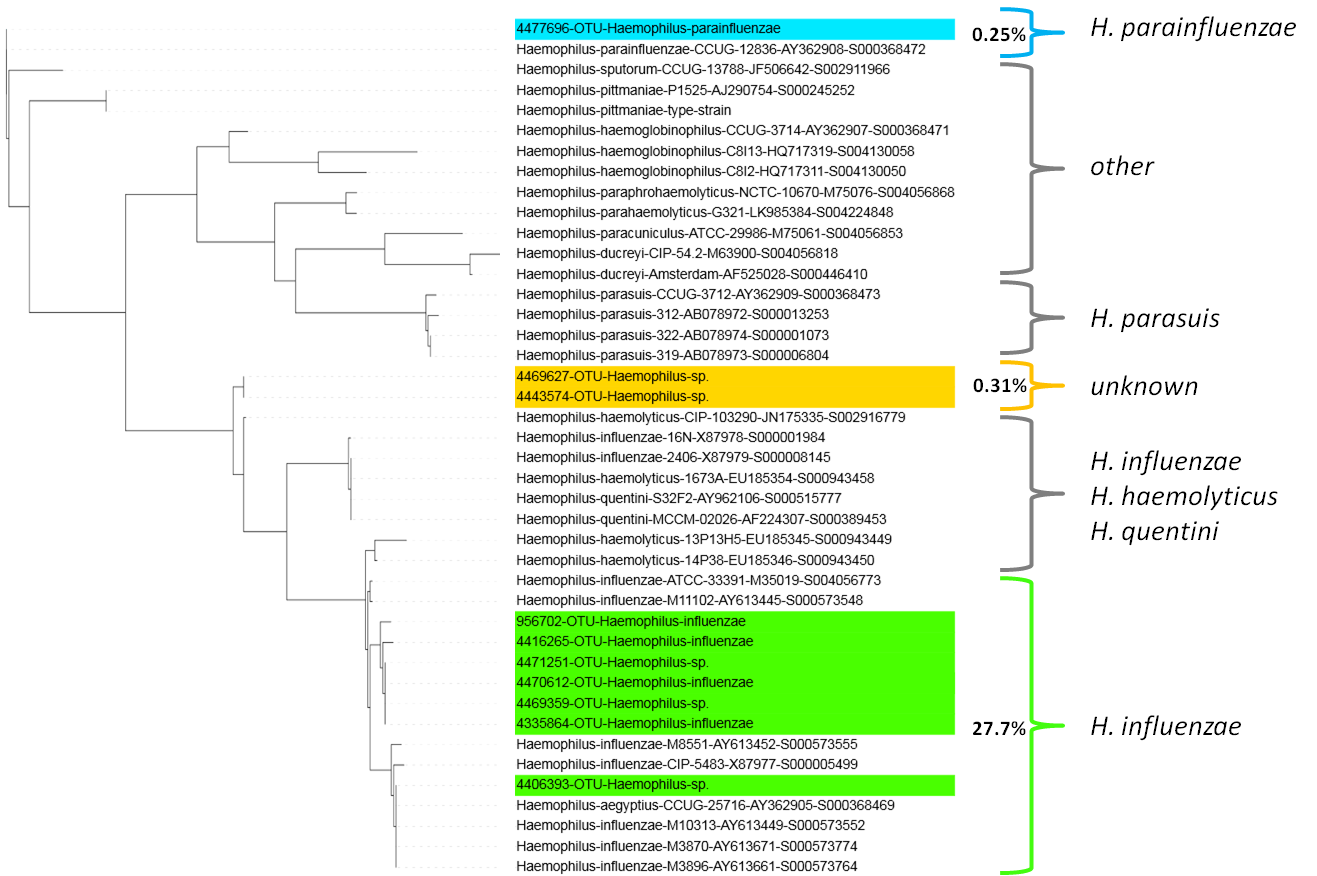
**

**Figure S7: Comparison of OTUs from current study to *Haemophilus* reference species shows that *Haemophilus*-classified OTUs are predominantly belonging to *Haemophilus influenzae* species*.*** 16S rRNA gene reference sequences from major *Haemophilus* species were selected from the RDP database [E1], and together with *Haemophilus*-classified OTU from current study (average relative abundance >0.01%, n = 10) an *in silico* PCR was performed with PrimerProspector [E2] on the V3-V4 region with primers as used in this study. The retrieved sequences were aligned by MUSCLE [E3], and a phylogenetic tree was build with FastTree [E4]. Newick file was uploaded to iTOL [E5], and OTU were manually colored for, based on above shown phylogenetic tree clusters, belonging to *H. parainfluenzae* (blue), *H. influenzae* (green) or another more distant, unknown cluster (yellow). From this analysis, we can conclude that OTU classified to the genus of *Haemophilus* predominantly belong to the species of *H. influenza,* also those classified to the *Haemophilus* genus only*,* with exception of OTU 4477696 which was correctly classified by our pipeline as *H. parainfluenzae* (with 0.25% average relative abundance), and 4469627 and 4443574, which belong to an unknown cluster (with together 0.31% average relative abundance)*.*

**References Supplementary Figures**

E1. Wang, Q., et al., *Naive Bayesian classifier for rapid assignment of rRNA sequences into the new bacterial taxonomy.* Appl Environ Microbiol, 2007. **73**(16): p. 5261-7.

E2. Walters, W.A., et al., *PrimerProspector: de novo design and taxonomic analysis of barcoded polymerase chain reaction primers.* Bioinformatics, 2011. **27**(8): p. 1159-61.

E3. Edgar, R.C., *MUSCLE: multiple sequence alignment with high accuracy and high throughput.* Nucleic Acids Res, 2004. **32**(5): p. 1792-7.

E4. Price, M.N., P.S. Dehal, and A.P. Arkin, *FastTree 2 – Approximately Maximum-Likelihood Trees for Large Alignments.* PLoS ONE, 2010. **5**(3): p. e9490.

E5. Letunic, I. and P. Bork, *Interactive Tree Of Life (iTOL): an online tool for phylogenetic tree display and annotation.* Bioinformatics, 2007. **23**(1): p. 127-8.
