# Supplementary material for: Haemophilus is overrepresented in the nasopharynx of infants hospitalized with RSV infection and associated with increased viral load and enhanced mucosal CXCL8 responses
Source: Microbiome. 2018 Jan 11;6:10. doi: 10.1186/s40168-017-0395-y (PMC5765694; doi:10.1186/s40168-017-0395-y)
Supplement: Supplementary file 6 — Supplementary information on methods. (DOCX 20 kb) [file 40168_2017_395_MOESM6_ESM.docx]

**Supplementary Methods**

**Study design**

This study was performed in two hospitals in Nijmegen, Radboud University Medical Center and Canisius Wilhelmina Ziekenhuis (CWZ). From the area of Nijmegen, children younger than 2 years of age with laboratory confirmed RSV infections were prospectively included during three consecutive winter seasons (2010/2011, 2011/2012 and 2012/2013), if they were hospitalized to the pediatric ward or intensive care unit (PICU). Written informed consent was obtained from all parents. Because the peak incidence of (severe) bronchiolitis is below the age of 6 months, and to limit the variation in age-related effects on microbiome and inflammatory response, we only included the children younger than 6 months with a PCR confirmed RSV infection. Healthy age-matched controls admitted to the hospital for surgery, who needed an elective inguinal hernia correction and had no signs of respiratory infection and a negative PCR for RSV, were included. We obtained permission from our medical ethical commission to collect control samples during one year, resulting in 21 samples as used in this study. Patients younger than 6 months with PCR confirmed RSV-positive bronchiolitis were selected and divided into three groups. Children without hypoxemia were classified as ‘mildly ill’, ‘moderately ill’ children received supplemental oxygen, while ‘severely ill’ children required mechanical ventilation. Patients with congenital heart or lung disease, immunodeficiency or glucocorticoid use were excluded. Within 24h after admission, a nasopharyngeal aspirate (NPA) was collected and parents from hospitalized children were asked for permission to collect a second NPA sample 4–6 weeks after admission (recovery). The final study cohort as reported here existed of n = 21 healthy infants, and n = 9 mild (2), n = 27 moderate (16) and n = 18 severe (7) patients (recovery samples in brackets). For more details on cohort design, available demographics and sample characteristics we refer to Additional file 1: Table S1.

The study was approved by the Central Committee on Research Involving Human Subjects of the Radboud University Medical Center.

**Sample collection**

The nasopharyngeal aspirates (NPA) were collected by introducing a catheter, connected to a collection tube and an aspiration system, into the nasopharyngeal cavity. Then, 0.5 ml of saline was instilled into the catheter and, while slowly retracting the catheter, the nasopharyngeal fluid was aspirated in a collection tube. Afterwards the catheter was flushed with 1 ml of saline and this was added to the collection fluid. Samples were kept cold and were immediately transferred to the laboratory. Samples were taken for viral and bacterial diagnostics. For viral diagnostics samples were analyzed by multiplex PCR, quantifying 15 different viral pathogens: influenza virus types A and B, coronavirus 229E and OC43, human bocavirus, enterovirus, adenovirus, parechovirus, PIV types 1–4, human metapneumovirus, rhinovirus (RV), and RSV, as previously described [E1]. See Additional file 1: Table S1B for co-infection information of the samples. The remaining NPA was centrifuged at 500 x *g* for 10 min at 4°C to spin down the mucus and cells, after which the supernatant was frozen at −80°C for ELISA.

**Measurement of cytokine and chemokine responses**

MMP-9, CXCL10, CCL5, IL6 and CXCL8 concentrations were measured in the nasopharyngeal aspirates by commercial ELISA kits (the first three from R&D Systems, the last two from Sanquin) according to the manufacturer’s instructions. MMP-9, CXCL10, CCL5, IL6 and CXCL8 had a detection limit of respectively 156 ng/ml, 1.56 ng/ml, 15.6 pg/ml, 156 pg/ml and 156 pg/ml. Samples below detection limit (<Min in Additional file 1: Table S1) were set to the sample value with lowest measured concentration for that cytokine.

**Bacterial DNA extraction**

NPA (300 μl) was resuspended in 343 μl lysis buffer (AGOWA Mag Mini DNA Isolation Kit, AGOWA) with 57 μl protease. Then, 25–50 mg sterile zirconium beads were added and 500 μl phenol. The samples were disrupted using the TissueLyser (Qiagen) for 2 min, twice. The samples were then centrifuged for 10 min at 10,000 rpm and the supernatant containing the released DNA was then purified according to the protocol included in the AGOWA Mag Mini DNA Isolation Kit, as described previously [E2]. Samples were resuspended in 50 μl elution buffer and stored at −80°C until further use.

**Bacterial DNA quantification and sample selection**

We determined the bacterial DNA concentration in each eluate by qPCR on the 16S rDNA gene, in order to correct for the subsequent 16S rRNA PCR DNA input material. The primer and probe sequences were as follows: forward primer 16S-UniF1 (764–782) 5′-CGA AAG CGT GGG GAG CAA A-3′; reverse primer 16S-UniR1 (894–879) 5′-GTT CGT ACT CCC CAG GCG G-3′; probe 5′-(FAM)-ATT AGA TAC CCT GGT AGT CCA-(MGB)-3′ as previously published by Bogaert *et al.*, 2011 [E3]. The 25 μl PCR mix was 1× TaqMan Universal PCR Master Mix, 10 μM of each primer (1 μl), 5 μM probe (1 μl), 6.5 μl DNA-free water, and 3 μl template DNA. Thermal cycling was performed in an ABI 7500 Fast Real-Time PCR System (cat. no. 4351107, Life Technologies, Carlsbad, CA, USA), with the following cycling conditions: 2 min 50°C, 10 min 95°C, and 50 cycles of 15 sec at 95°C and 1 min at 65°C. The 16S rDNA standard curve consisted of a 10-fold dilution series of a mix of genomic DNA extracted from three bacteria common to the respiratory tract: Streptococcus pneumoniae (TIGR4), Moraxella catarrhalis (RH4), and Haemophilus influenzae (1521062).

We extracted genomic DNA with the Qiagen Genomic-tip 20/G Kit (cat. no. 10223, Qiagen, Venlo, The Netherlands) and quantified it by a spectrophotometer (NanoDrop ND-1000, Thermo Fisher Scientific, Wilmington, DE, USA). Those participants whose extracted DNA samples all contained at least 1 pg/μl of bacterial DNA were considered eligible for microbiome analysis [E4]. To avoid false-positive results, during DNA extraction as well as during amplification procedures negative controls were included.

**16S rRNA gene amplification prior to sequencing**

To generate the PCR amplicon libraries, sample-specific barcoded amplicons for the V3-V4 hypervariable region of the small subunit ribosomal RNA 16S genes were generated using a two-step PCR. 10-25 ng genomic (g)DNA was used as template for the first PCR with a total volume of 50 µl using the 341F (5’-CCT ACG GGN GGC WGC AG-3’) and the 785R (5’-GAC TAC HVG GGT ATC TAA TCC-3’) primers appended with Illumina adaptor sequences.

**16S rRNA marker gene sequencing**

Illumina 16S rRNA amplicon libraries were generated at BaseClear BV (Leiden, The Netherlands). The libraries were checked on a Bioanalyzer (Agilent Technologies, Waldbronn, Germany) and quantified. The libraries were multiplexed, clustered and sequenced on an Illumina MiSeq system with paired-end 300 cycles protocol and indexing. The sequencing run was analyzed with the Illumina CASAVA pipeline (v1.8.3) with demultiplexing based on sample-specific barcodes. The raw sequencing data produced was processed removing the sequence reads of too low quality (only "passing filter" reads were selected) and discarding reads containing adaptor sequences or failing PhiX Control with an in-house filtering protocol. A quality assessment on the remaining reads was performed using the FASTQC quality control tool version 0.10.0.

**Illumina sequencing data analysis**

Multiplexed FASTQ files as provided by BaseClear were first used to generate Illumina paired-end sequence pseudoreads by PEAR [E5], using the default settings. For gene sequencing analysis, a customized Python workflow based on Quantitative Insights Into Microbial Ecology (QIIME version 1.8) [E6] was adopted (<http://qiime.org>). Reads were filtered for chimeric sequences using the UCHIME algorithm version 4 [E7]. Hierarchical clustering of samples was performed using UPGMA with weighted UniFrac as a distance measure as implemented in QIIME 1.8. Figures resulting from these clustering analyses were generated using the interactive tree of life (iTOL) tool [E8]. The Ribosomal Database Project classifier version 2.3 was performed for taxonomic classification of the sequence reads [E9]. Alpha diversity metrics (PD whole tree, Chao1, Observed Species and Shannon) were calculated by bootstrapping 4822 reads per sample, and taking the average over 10 trials. Chao1 is a species richness metric; whereas PD whole tree is a species diversity metric (defined as a combination of both species richness and species evenness) comparable to Shannon but additionally taking taxonomical distance into account. For visualization of the differential microbiome, Cytoscape software version 3.1.3 [E10] was used together with in-house developed Python scripts for generating the appropriate input data deriving from the QIIME analysis.

Note that due to technical limitations in the resolution of 16S marker gene sequencing, OTU (Operational Taxonomic Unit) calling on the level of species should be interpreted with caution.

**Microbiota sequencing data availability**

The raw, unprocessed 16S rRNA marker gene Illumina sequencing reads are publicly available for download at the European Nucleotide Archive (ENA) database (<http://www.ebi.ac.uk/ena>) under study accession number PRJEB20811 (or secondary accession number ERP022997) [E11]. The sequencing data is available in FASTQ-format, including corresponding metadata for each sample.

**Statistics**

For the microbiota data in this manuscript, statistical significance between contrasts with regard to taxonomy abundances was tested by a non-parametric (unpaired) Mann-Whitney U (MWU), corrected with False Discovery Rate (FDR) for multiple testing; unless stated otherwise. Statistical tests were performed by custom, in-house Python scripts (SciPy module version 0.17.0; https://www.scipy.org/). Multivariate Redundancy Analysis (RDA) and Principal Component Analysis (PCA) was done using Canoco 5.04 [E12] using default settings of the analysis type ‘Constrained’ or ‘Unconstrained’, respectively. In addition, log transformation was set to 100, unless stated otherwise. To prevent undesired biases in the multivariate RDA and PCA analyses, age, gender and birth weight was always taken along as a covariate (Additional file 2: Figure S1). Relative abundance values for taxa were used as response data, and in case of RDA, the sample RSV status as explanatory variables. RDA calculates *p-*values by randomly permuting the sample RSV status, and by thereafter counting the number of times that a permuted set of samples had a better separation than the original. For all other experimental data (i.e. protein measurements, metadata, etc.), statistical significance was tested likewise using a non-parametric Kruskal–Wallis one-way ANOVA, with Dunn's correction for multiple testing (GraphPad Prism 5.0); unless stated otherwise. Correlations were examined using Spearman’s rank test as performed by custom, in-house R scripts (version 3.2.2; <https://www.r-project.org/>). Significances mentioned in figures are as follows: *n.s.* (not significant), * *p* < 0.05, ** *p* < 0.01, *** *p* < 0.001.

**References Supplementary Methods**

E1. Templeton, K.E., et al., *Rapid and sensitive method using multiplex real-time PCR for diagnosis of infections by influenza a and influenza B viruses, respiratory syncytial virus, and parainfluenza viruses 1, 2, 3, and 4.* J Clin Microbiol, 2004. **42**(4): p. 1564-9.

E2. Shak, J.R., et al., *Impact of experimental human pneumococcal carriage on nasopharyngeal bacterial densities in healthy adults.* PLoS One, 2014. **9**(6): p. e98829.

E3. Bogaert, D., et al., *Variability and diversity of nasopharyngeal microbiota in children: a metagenomic analysis.* PLoS One, 2011. **6**(2): p. e17035.

E4. Biesbroek, G., et al., *Deep sequencing analyses of low density microbial communities: working at the boundary of accurate microbiota detection.* PLoS One, 2012. **7**(3): p. e32942.

E5. Zhang, J., et al., *PEAR: a fast and accurate Illumina Paired-End reAd mergeR.* Bioinformatics, 2014. **30**(5): p. 614-20.

E6. Caporaso, J.G., et al., *QIIME allows analysis of high-throughput community sequencing data.* Nat Methods, 2010. **7**(5): p. 335-6.

E7. Edgar, R.C., et al., *UCHIME improves sensitivity and speed of chimera detection.* Bioinformatics, 2011. **27**(16): p. 2194-200.

E8. Letunic, I. and P. Bork, *Interactive Tree Of Life (iTOL): an online tool for phylogenetic tree display and annotation.* Bioinformatics, 2007. **23**(1): p. 127-8.

E9. Wang, Q., et al., *Naive Bayesian classifier for rapid assignment of rRNA sequences into the new bacterial taxonomy.* Appl Environ Microbiol, 2007. **73**(16): p. 5261-7.

E10. Shannon, P., et al., *Cytoscape: a software environment for integrated models of biomolecular interaction networks.* Genome Res, 2003. **13**(11): p. 2498-504.

E11. Leinonen, R., et al., *The European Nucleotide Archive.* Nucleic Acids Res, 2011. **39**(Database issue): p. D28-31.

E12. ter Braak, C.J.F. and Smilauer P., *Canoco reference manual and user's guide: software for ordination, version 5.0*. Ithaca USA. Microcomputer Power - **496**: p. 201
